# Improvement in right ventricular function during reversibility testing in pulmonary arterial hypertension: a case report

**DOI:** 10.1186/1476-7120-7-9

**Published:** 2009-02-19

**Authors:** Sandrine Huez, Jean-Luc Vachiéry, Robert Naeije

**Affiliations:** 1 Department of Cardiology, Hôpital Erasme, Université Libre de Bruxelles, Bruxelles, Belgium; 2 Department of Pathophysiology, Erasmus Campus, CP 604, Route de Lennik 808, B-1070 Brussels, Belgium

## Abstract

A right heart catheterization with reversibility testing is recommended for the diagnosis and treatment of pulmonary arterial hypertension. In this 24 years-old woman, the inhalation of 5 μg iloprost transiently decreased mean pulmonary artery pressure from 62 to 36 mmHg and pulmonary vascular resistance from 11.0 to 4.9 Wood units, meeting the criteria of a "positive response". The echocardiographic examination showed normalization of right heart chamber dimensions and of the right ventricular performance (Tei) index. Pulsed tissue Doppler imaging of the right ventricle showed a decrease in the isovolumic relaxation time from 102 to 73 ms, and an increase of the E/A ratio from 0.72 to 1.38, together with marked improvements in mid-apical free wall systolic strain and strain rate. A positive response to reversibility testing of pulmonary arterial hypertension may be associated with quasi normalization of right ventricular function, in spite of still elevated pulmonary artery pressure.

## Introduction

The diagnosis of pulmonary arterial hypertension (PAH) relies on a right heart catheterization, with reversibility testing to identify patients who might benefit from calcium channel blocker therapy [1]. A positive response is defined by a decrease in mean pulmonary artery pressure (Ppa) by at least 10 mmHg to below 40 mmHg, with unchanged or increased cardiac output (Q), with the inhalation of nitric oxide (NO) or infusion of epoprostenol or adenosine [1]. Treatment of these "responders" with calcium channel blockers may result in sustained clinical benefit [2]. A logical explanation for these beneficial clinical effects is an improved right ventricular function (RV) because of a decreased afterload. However, little is known about the effects of reversibility testing on RV function.

We therefore report the evaluated RV function with Doppler echocardiography and pulse tissue Doppler imaging in a case of a patients with idiopathic PAH who presented with a borderline response to inhaled NO and a more definitive response to the inhaled prostacyclin analogue iloprost.

## Case presentation

This 24 years-old woman with idiopathic PAH had been referred for right heart catheterization and reversibility testing. The results are shown in Table 1. The "responder" criteria were almost met with the inhalation of 20 ppm of NO, and definitively with the inhalation of 5 μg of iloprost, which furthermore decreased PVR by more than 50%.

**Table 1:** Hemodynamic measurements at baseline and during the inhalation of 20 ppm nitric oxide (NO) and 5 μg of iloprost.

Variables	Baseline	Inhaled NO	Iloprost
Ppa, mmHg	62	45	36

Ppao, mmHg	9	11	10

Pra, mmHg	7	6	7

Q, L/min	4.8	4.5	5.3

PVR, WU	11.0	7.6	4.9

Abbreviations: Ppa = mean pulmonary artery pressure. Ppao = pulmonary occluded pulmonary artery pressure. Pra = right atrial pressure. Q = cardiac output. PVR = pulmonary vascular resistance.

An echocardiography examination with tissue Doppler imaging (TDI) was performed as previously reported [3]. Mean Ppa was estimated from the acceleration time of pulmonary flow, measured at the RV outflow tract [4]. Right ventricular function was assessed by measurements of the area shortening fraction, the RV to left ventricular (LV) end-diastolic area ratio (4 chamber apical view) and the LV eccentricity index (parasternal short axis view) [5]. A index of global RV function was measured as reported by Tei et al (the "Tei index") [6]. Pulsed TDI was applied to measure mitral and tricuspid annuli peak velocities during systole (S), early diastole (E) and late diastole (A) [7]. The isolvolumic relaxation time (IVRT) was measured as the time between the end of the S and beginning of the E waves. Strain and strain rate measurements were obtained in 2 operator selected regions of interest, the mid-basal and the mid-apical segments of the free RV wall [3]. All the measurements were obtained before and during the reversibility tests.

**Figure 1:**
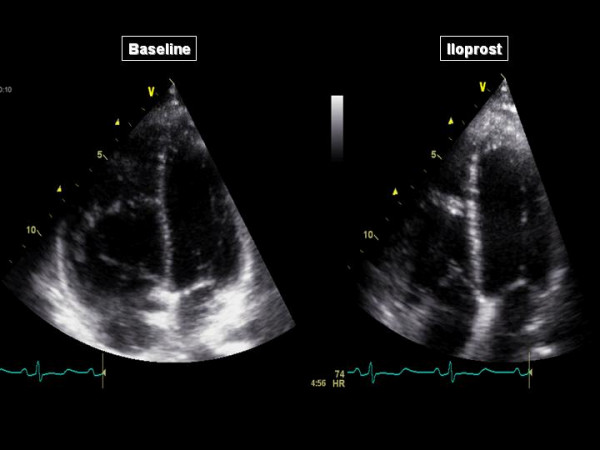
**Four chamber apical view before (left) and during iloprost inhalation (right)**.

**Figure 2:**
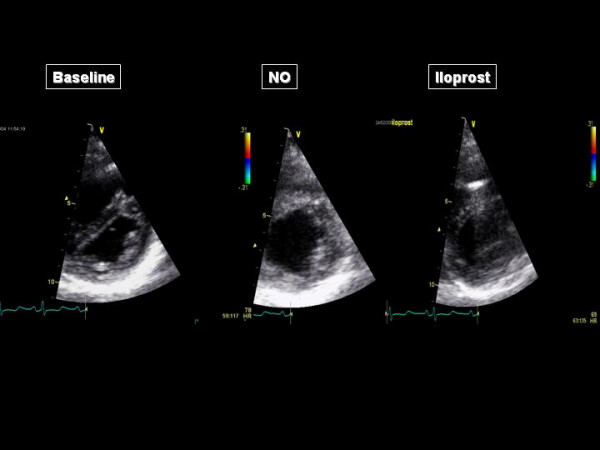
**Parasternal short axis view before (left), during nitric oxide (NO) inhalation (middle) and during iloprost inhalation (right)**.

**Figure 3:**
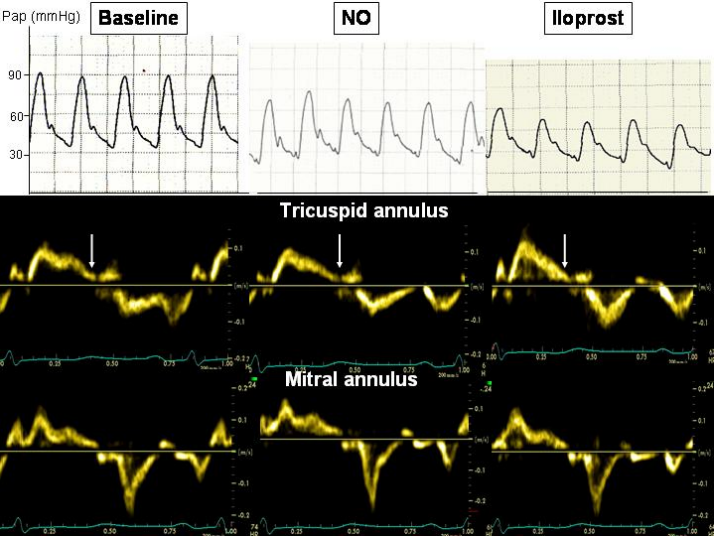
**Pulmonary artery pressure (top) and pulsed-tissue Doppler traces at the tricuspid annulus (middle) and at the mitral annulus (at the bottom) before and during the inhalation of iloprost**. The white arrows show the pulmonary valve closure.

**Figure 4:**
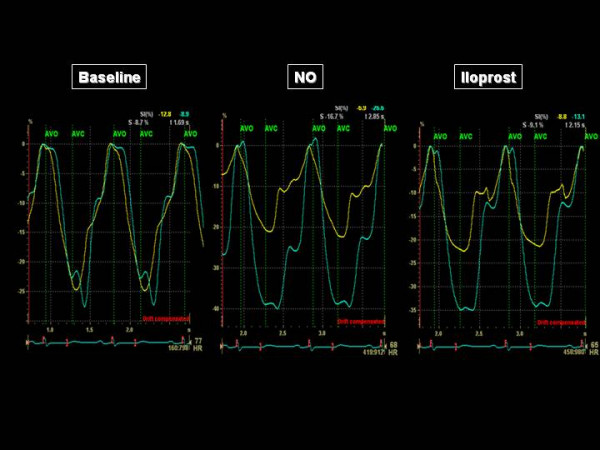
**Systolic strain recorded along the right ventricular free wall, at the mid-base (yellow trace) and at the mid-apex (green trace), before and during reversibility testing**. AVO indicates the pulmonary valve opening and AVC the pulmonary valve closure.

**Figure 5:**
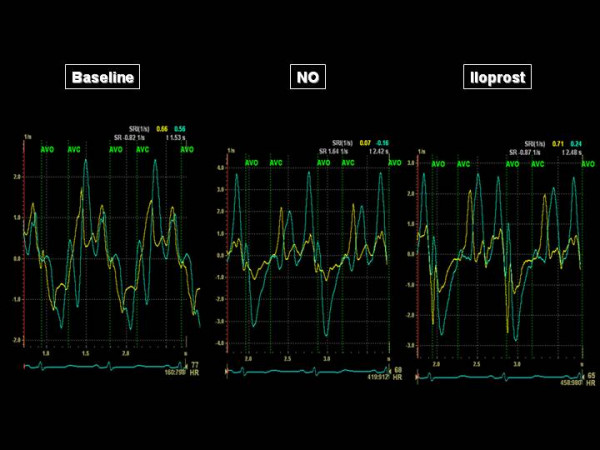
**Systolic strain rate recorded along the right ventricular free wall, at the mid-base (yellow trace) and at the mid-apex (green trace), before and during reversibility testing**. AVO indicates the pulmonary valve opening and AVC the pulmonary valve closure.

The acceleration time of pulmonary blood flow changed from 80 ms to 81 ms (NO) and 103 ms (iloprost), indicating a decrease in Ppa that was more important with iloprost than with NO. The four chamber apical view showed that the RV came back to a near-normal shape, with a RV/LV end-diastolic area ratio decreased from 91 to 54% with iloprost (Figure 1). The short axis view showed a return of the LV to a normal rounded shape, with an eccentricity index in end-diastole: from 1.8 to 1.1 (NO) and to 1 (iloprost) and in end-systole from 2.3 to 1.2 (NO) and to 1.1 (iloprost) (Figure 2). The RV area shortening fraction increased from 11 to 43% with iloprost, and the RV Tei index was normalized, from 0.31 to 0.14 (NO) and to 0.16 (iloprost). Pulsed TDI recorded at the tricuspid annulus showed a marked increase in S waves, from 11 to 12 and 13.5 cm/sec, and in E waves, from 8 to 9.5 and to 11 cm/sec and a decrease in A wave (from 11 to 8.5 and to 8 cm/sec) with inversion of the E/A ratio (from 0.72 to 1.12 to 1.38), and a decrease in IVRT (from 102 to 75 and to 73 msec), whereas these effects were not observed at the mitral annulus (Figure 3). Along the RV free wall, systolic strain increased at the mid-apex, from 19 to 41 and to 37%, but not at the mid-base, from 23 to 23 and to 22%. The changes in systolic strain rate were similar, with at mid-apex from 1.1 to 3.2 and to 3.6/sec and at mid-base from 1.1 to 1.1 and 1.1/sec (Figures 4 and 5).

Because the criteria of reversibility were met [2], the patient was initially treated with calcium channel blockers. This treatment was poorly tolerated, and soon replaced by the endothelin receptor antagonists sitaxsentan, which resulted in a stabilization in New York Heart Association functional class II and an acceptable quality of life.

## Discussion

The present results show that all echocardiographic indices of both systolic and diastolic RV and LV function returned to normal or near-normal during a positive reversibility test, in spite of the fact that Ppa remained higher than normal.

The symptomatology and prognosis of PAH are largely determined by RV function adaptation. Accordingly, right atrial pressure and cardiac output have been shown to be better predictors of outcome than the actual level of Ppa [8]. Recent studies have drawn attention to the importance of RV systolic function adaptation [9]. End-systolic elastance as a measure of contractility has been shown to be increased in idiopathic PAH, though not enough to match increased afterload as measured by arterial elastance [10]. Accordingly, the RV is chronically uncoupled in PAH, accounting for inability to increase flow output in response to peripheral demand by the exercising muscles. However, chronically increased systolic function may allow for an only partially unloaded RV to normalize its coupling to the pulmonary circulation, explaining the marked improvement of indices of RV function in the present case. In addition, our results are in keeping with the notion that abnormal RV and LV diastolic changes in PAH are secondary to systolic RV failure.

It is if interest that TDI indices of systolic function changes were essentially mid-apical, confirming a previous report in a PAH patient with abrupt decrease in PVR after a double lung transplantation [11]. While still unexplained, this observation underscores the need of regional function measurements, which are possible with TDI, for the complete description of RV function changes.

The decrease in Ppa and PVR in the present PAH patient was more important with inhaled iloprost than with inhaled NO, so that the "responder" criteria were actually only met with inhaled iloprost. Inhaled iloprost is not recommended for reversibility testing [1], but it has been reported previously to be a more potent pulmonary vasodilator intervention than inhaled NO in PAH patients [12].

## Conclusion

Decreased Ppa and PVR during reversibility testing in PAH may be associated with a quasi-normalization of RV function, accounting for clinical improvement even if pulmonary hemodynamics are not completely reversed back to normal.

## Consent

Written informed consent was obtained from the patient of this case report. A copy of the written informed consent is available for review by the Editor-in-Chief of this journal.

## Competing interests

The authors declare that they have no competing interests.

## Authors' contributions

SH performed the echocardiographic examination and JLV the right heart catheterization. RN wrote the report. SH, JLV and RN were involved in the patient's clinical care. All authors read and approved the manuscript.
